# Polymer-Based Drug Delivery Systems for Cancer Therapeutics

**DOI:** 10.3390/polym16060843

**Published:** 2024-03-19

**Authors:** Ling Ding, Prachi Agrawal, Sandeep K. Singh, Yashpal S. Chhonker, Jingjing Sun, Daryl J. Murry

**Affiliations:** 1 Clinical Pharmacology Laboratory, Department of Pharmacy Practice and Science, University of Nebraska Medical Center, Omaha, NE 68198, USA; ling.ding@unmc.edu (L.D.); sasingh@unmc.edu (S.K.S.); y.chhonker@unmc.edu (Y.S.C.); 2 Department of Pharmaceutical Sciences, University of Nebraska Medical Center, Omaha, NE 68198, USA; pagrawal@unmc.edu (P.A.); jsun@unmc.edu (J.S.); 3 Fred and Pamela Buffett Cancer Center, University of Nebraska Medical Center, Omaha, NE 68198, USA

**Keywords:** natural/synthetic polymer, polymeric drug, biodegradable/bioabsorbable, drug delivery systems

## Abstract

Chemotherapy together with surgery and/or radiotherapy are the most common therapeutic methods for treating cancer. However, the off-target effects of chemotherapy are known to produce side effects and dose-limiting toxicities. Novel delivery platforms based on natural and synthetic polymers with enhanced pharmacokinetic and therapeutic potential for the treatment of cancer have grown tremendously over the past 10 years. Polymers can facilitate selective targeting, enhance and prolong circulation, improve delivery, and provide the controlled release of cargos through various mechanisms, including physical adsorption, chemical conjugation, and/or internal loading. Notably, polymers that are biodegradable, biocompatible, and physicochemically stable are considered to be ideal delivery carriers. This biomimetic and bio-inspired system offers a bright future for effective drug delivery with the potential to overcome the obstacles encountered. This review focuses on the barriers that impact the success of chemotherapy drug delivery as well as the recent developments based on natural and synthetic polymers as platforms for improving drug delivery for treating cancer.

## 1. Introduction

Cancer represents a significant global public health challenge, ranking as the second leading cause of death worldwide, following cardiovascular disease, which are the primary causes of mortality on the global scale [1]. In 2023, over 1.9 million new cancer cases were projected to be diagnosed in the United States, with approximately 609,820 deaths [2]. Chemotherapy in combination with surgery and/or radiotherapy are the most common therapeutic approaches for cancer treatment. Chemotherapy often relies on the use of small toxic chemotherapeutic molecules that can interfere with the DNA and impact the cellular macromolecular synthesis. However, these treatments are known to produce side effects due to their non-selective and off-target effects [3]. Advancements in the understanding of cancer biology have paved the way for the development of monoclonal antibodies (mAbs) as promising cancer therapeutics. Notably, rituximab and trastuzumab were the first-approved mAb therapeutics for the treating of lymphoma and breast cancer, respectively (in 2011 by the U.S. FDA) [4]. mAbs have the unique capability to target tumor cells directly, while also simultaneously enhancing the body’s immune response against the cancer. Furthermore, mAbs are being explored in various strategies for cancer therapy, including polymer-based approaches for improving T-cell infiltration and anti-PD-L1 immunotherapy [5]. These polymer-based innovative approaches represent significant progress in cancer treatment, offering potential therapeutic benefits while minimizing side effects.

The effectiveness of an anticancer agent depends on its ability to accumulate at an effective concentration at the target site, and numerous innovative drug delivery approaches have been developed and advanced to clinical trials. Targeted drug delivery systems require that the delivery vehicle and drug work together to fulfill their therapeutic objective, ensuring that the therapeutics reach the right sites at effective concentrations and engage the target for the appropriate duration. Drug delivery technologies have played a pivotal role in creating pharmaceutical formulations that improve patient health by enhancing the target site delivery and promoting patient compliance. Over the past two decades, drug delivery systems (DDSs) have undergone great development. These systems rely on improving the bioavailability and therapeutic efficacy of active pharmaceutical ingredients (APIs), while minimizing off-target effects. DDSs are typically designed with three primary objectives: (1) enhance the solubility and stability of the APIs, (2) bring pharmacological effects to the targeted site, and (3) minimize the off-target and dose-limiting side effects. Polymeric DDSs have rapidly progressed since the 1980s and are engineered in increasingly specific ways, offering a more personalized approach to drug delivery. Generally, polymeric DDSs can be classified as nanoparticulate delivery systems and polymer–drug conjugates (PDCs). Particulate delivery systems, considered as the APIs, are encapsulated into NPs physically and include micelles, nanogels, polymeric hybrid nanoparticles (NPs), and non-covalent polymeric carrier systems, whereas PDCs are focused more on conjugating with cell-specific antigens or receptors. Coupling low-molecular-weight (LMW) APIs with high-molecular-weight (HMW) polymers via a cleavable linker is an effective method for improving anticancer efficacy. N-(2-hydroxypropyl)metharylamide (HPMA) conjugates of doxorubicin (DOX), paclitaxel (PTX), camptothecin (CPT), and platinum (II) complexes were the first candidates evaluated in clinical trials; since then, several PDC candidates have been investigated in clinical trials [6]. Polymeric NPs can be optimized by the coupling of targeting/responsive ligands, thereby increasing the selectivity for cancer cells while improving the intracellular drug delivery, reducing off-target effects, side effects, and drug toxicity. Importantly, polymeric systems have played a vital role in enhancing the effectiveness and safety profile of immunotherapy in the context of antitumor treatments [7]. However, a well-known concern related to polymer-based DDSs is the uncertainty with regard to safety, particularly in relation to immune responses. Furthermore, considerations associated to the drug-loading capacity, stability, and manufacturing challenges are pivotal aspects that must be carefully addressed when designing an effective delivery system.

Polymer-based DDSs have garnered considerable attention over the past two decades, because they offer the ability to specifically target tumor tissues and significantly improve the therapeutic window of the therapeutic. For example, poly (lactic glycolic acid) (PLGA) is a biodegradable polymer that has been approved by the U.S. FDA, and several PGLA-based formulations have received clinical approval, including Decapeptyl, Suprecur MP, Lupron Depot, among others [8]. Polymer-based DDSs have been defined as delivery systems or formulations based on polymers that can introduce therapeutics into the body, with a primary aim of enhancing bioavailability, safety, and efficacy. The use of polymer-based DDSs is the most effective strategy for overcoming the limitations of chemotherapy for cancer treatment. They achieve this by regulating the rate of drug release and directing the drugs to specific biological sites. One critical aspect of polymer-based DDSs is the use of biodegradable and bioabsorbable polymers, which provide a safe framework for delivering therapeutics without harm to the body [9]. Natural polymers, including chitosan, arginine, cyclodextrin, cellulose, hyaluronic acid (HA), and polysaccharides, have been employed for many years to enhance drug delivery efficacy [10]. These natural polymers have undergone modifications to produce novel biomaterials for controlled drug delivery applications. Additionally, synthetic polymers are of growing interest due to the ease of their structure modification and their bio-responsive and bioactive features. Aliphatic polyesters, such as poly (ethylene glycol) (PEG), poly (glycolic acid) (PGA), poly (lactic acid) (PLA), or PLGA, poly (caprolactone), and polydioxanone, are the most commonly used ingredients in DDSs [8]. Hydrogels prepared from bioabsorbable polymers, such as PLA and PLGA, and their copolymers have been employed to create the delivery cancer therapeutics for improving drug delivery and efficacy [11]. In addition, PEG stands out as the most powerful approach to address issues related to low solubility and metabolic stability. Upon the PEGylation, PEG forms a stable hydration layer, leading to an increased blood circulation time and metabolic stability, while reducing the immunogenicity by hindering the approach of plasma proteins and macrophages, which is called the “stealth effect” [12]. However, it is important to mention that PEGylation also suffer from drawbacks, such as decreasing the bioactivity and formation of anti-PEG antibodies that may accelerate blood clearance. Pegfilgrastim (Neulasta^®^), the first product of PEG chemistry, was approved in 2002, which is a granulocyte-colony-stimulating factor (G-CSF) modified with a 20-kDa linear PEG at its N-terminus [13]. PEGylated formulations of several drugs, such as PEGylated liposomal DOX, have received FDA approval for cancer treatment [14]. A comprehensive review of PEGylation approaches to synthetic DDSs can be found in [12].

The use of natural biodegradable materials in medicine has a rich history dating back to ~3000 BC, when ancient Egyptians used plant fibers and wool fibers as suture materials [9]. These degradable polymers can break down inside the body, producing non-toxic natural byproducts, like water and carbon dioxide, which are easily eliminated [9]. Degradable polymers often incorporate labile ester, anhydride, and amide chemical linkages, resulting in the degradation of polymer chains [15]. Several features are required for a PDCs to be both effective and practical, including being non-toxic and non-immunogenic. Additionally, the molecular weight should be high enough to ensure long circulation times, but lower than 40 kDa to ensure renal elimination following drug release [16].

While significant achievements in drug delivery have occurred over the past two decades, the challenges of regulating drug entry into tumor tissues remain. The ultimate goal of polymer-based DDSs is to achieve effective therapeutic concentrations at the precise biological site. This review offers a detailed discussion of recent advances in polymer-based DDSs for cancer therapy. In this review, we highlight the barriers that impact the cancer therapeutics for reaching to the biological sites; specifically, natural and synthetic polymers for DDSs are comprehensively discussed.

## 2. Barriers That Impact the Therapeutics Reaching the Tumor Sites

APIs can only be impactful if they help to achieve sufficient drug concentrations at the pharmacological target to obtain the desired therapeutic effect. However, a limited drug accumulation at the site of action may result in treatment failure or the development of drug resistance. Biological barriers are often critical to a successful therapeutic delivery. The identification and understanding of the barriers that impede drug delivery constitute the foremost and fundamental steps in the design of effective DDSs for overcoming the pharmacokinetic limitations associated with biological barriers. There are several biological barriers that significantly impact therapeutic effectiveness, including transporter barriers, mononuclear phagocyte systems (MPSs), vascular barriers, extracellular matrix (ECM) barriers, blood–brain barrier (BBB), cellular uptake and efflux barrier, immune system barrier, and the tumor microenvironment (TME) [17]. Transport barriers include MPSs, tumor vasculature, tumor endothelium, the ECM, and barriers within cancer cells [18].

### 2.1. TME Barriers

The TME refers to all the physiological and biochemical compositions around the tumor cells, which consist of tumor stroma, the ECM, and cells including the endothelial cells, cancer-associated fibroblasts (CAFs), activated stellate cells, mesenchymal stromal cells, chondrocytes, and immune-infiltrating cells [19]. The ECM consists of a cross-linked network of elastin fibers, fibronectin, collagens, and HA. This highly dense stroma around the tumor cells results in a significant resistance to the passive diffusion of therapeutics. Moreover, those cells crosstalk with ECM components, together providing the necessary environment for tumor progression and metastasis [20]. CAFs play an important role in the TME, which promotes tumor growth and invasion through various mechanisms. CAFs are activated in the tumor tissues and secrete various cytokines, such as vascular endothelial growth factor (VEGF), tumor necrosis factor (TNF), interleukin-6 (IL-6), transforming growth factor beta (TGF-β), hepatocyte growth factor (HGF), fibroblast-secreted protein-1 (FSP-1), and stroma-derived factor-1 (SDF-1) [21]. CAFs can also release some enzymes, such as matrix metalloproteases (MMPs). The crosstalk between CAFs and tumor cells is the most crucial pathway in tumor progression. These neoplastic cells together with the surrounding stromal cells dramatically secret abundant angiogenic growth factors, like VEGF and TNF, leading to an extensive abnormal vessel growth, tumor progression, and metastasis [22]. Moreover, the high interstitial fluid pressure (IFP) expressed in the tumor also limits therapeutic distribution and penetration into the deep recesses of the tumor, as the drugs are effluxed from the vasculature [23]. Hypoxia, acidity, and abnormally high IFP are also involved in the tumor metastasis, reduce the sensitivity of chemotherapy, and increase the development of drug resistance to chemotherapy [24]. The TME has a variety of enzyme expression patterns, including MMPs, polysaccharides, prostate-specific antigen (PSA), and indoleamine 2,3-Dioxygenase, which create effective enzyme-responsive nano-drug delivery systems. Various stimuli-responsive polymer-based DDSs have been developed to overcome these TME barriers for targeting drug delivery to tumor sites, for example, polymers that can modulate the TME, active tumor targeting by ligand modification and biomimetic strategies, and TME-response drug delivery. The details of stimuli-responsive polymer-based DDSs for cancer therapeutic delivery are reviewed in Section 3.

### 2.2. Other Biological Barriers

The majority of the presently employed, systemically administrated anticancer therapeutics depend on transvacuolar mechanisms for tumor penetration and accumulation. Nevertheless, passive trans-endothelial delivery is characterized by sluggish and inefficient kinetics and typically requires high drug concentrations across the tumor endothelial cell (EC) barriers [25]. Therefore, the bloodstream and ECs are the other major barriers for the successful anticancer therapeutics’ delivery. Additionally, absorption from the gastrointestinal (GI) tract, metabolic stability, and the BBB serves as another significant biological obstacle for the oral administration of anticancer therapeutics targeting brain neoplasms. Overcoming GI barriers is crucial for advancing strategies to enhance oral drug bioavailability. However, even after reaching the tumor site, the tumor cells and nuclear membranes as well as lysosome escape also play important roles in the in vivo drug delivery. Importantly, the carrier-mediated polymer-based DDSs may improve therapeutic delivery across the BBB for targeting the drug accumulation into brain.

Increased attention has been paid to transporters due to the continuous occurrence of multi-drug resistance in the clinic. Small therapeutics administrated orally are commonly regarded as substrates for one or more uptake transporters expressed in the intestines and liver. The efflux transport limits the exposure to potential therapeutic agents. Polymer-based DDSs have been developed to enhance either intestinal absorption or plasma concentration by increasing the drug solubility, mucosal permeability, and stability in the GI tract environment [16]. Utilizing transporter-based polymers can improve the targeted uptake of the drugs while inhibiting their efflux. For example, polymer-based strategies conjugated/coupled with ligands capable of binding to specific receptors on the brain microvascular endothelial cells (BMECs) may enhance BBB penetration by receptor-mediated transcytosis in BMECs [26].

## 3. Polymer-Based DDSs for Cancer Therapeutics

### 3.1. Natural Polymer-Based DDSs

Cancer patients frequently require chemotherapy, which comes with numerous drawbacks, such as toxicity to healthy tissues, lack of drug efficacy due to multi-drug resistance, and cost concerns. To mitigate these issues, polymer-based DDSs play a vital role in minimizing the disadvantages associated with the delivery of free xenobiotics. Amongst polymer-based DDSs, natural polysaccharides are highly favored due to their abundance in nature and renewability. Their non-toxic, water-soluble, biodegradable, and biocompatible properties offer pronounced advantages over synthetic polymers. Some of the most used natural polymers in the pharmaceutical industry are discussed below.

#### 3.1.1. Chitosan

**Figure 1:**
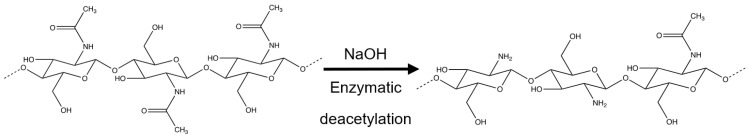
Structure of chitosan.

Chitosan, a biocompatible and biodegradable polysaccharide, is naturally derived through the N-deacetylation of chitin [27]. Its structure is closely related to that of cellulose and is most abundantly found in fungi and the exoskeleton of crustaceans [28,29]. Widely applicable across agriculture, food industry, and biomedical research, chitosan is a cationic linear polymer with repeating subunits of D-glucosamine and N-acetyl D-glucosamine linked by 1-4 glycosidic bonds (Figure 1). Chitosan is the only polycation in nature, and its charge density depends on the acetylation degree and pH. Chitosan is insoluble at alkaline and neutral pH values, but readily soluble in the presence of acids due to the protonation of amines [29]. Because of its cationic property, chitosan provides various biopharmaceutical properties, including mucoadhesion, transfection, and permeation enhancement [30]. Physicochemical properties, like solubility, vary with the degree of deacetylation (in the range of 5–95%) and molecular weight (50–20,000 kDa) [31,32,33]. Chitosan acts as a permeation enhancer irrespective of the degree of deacetylation on a low-molecular-weight polymer. The solubility of the polysaccharide is inversely proportional to the molecular weight. Furthermore, low-molecular-weight and less deacetylated chitosan can intensify the antibacterial effect against Gram-negative bacteria but decrease it against Gram-positive bacteria [32]. Therefore, the careful selection of an optimal-grade polymer is crucial for an effective drug delivery [33]. However, a high degree of deacetylation (DD) may lead to an increased risk of toxicity. The DD of chitosan is the amount of acetyl groups that have been removed from the chitosan structure, and a higher DD means that there are more available amine groups [34].

Chitosan NPs have been recognized as a promising drug delivery system for pharmaceuticals like antimicrobials, growth factors, anti-inflammatories, and anticancer agents. Beyond its role as a DDS in the form of NPs, there is a growing inclination towards using chitosan as a vehicle (gels) for the delivery of other carriers [35]. Primary techniques involved in the preparation of chitosan NPs or hydrogels include ionic gelation, ionic or covalent cross-linking, emulsion solvent diffusion, and precipitation, alone or in combination [28,33]. The following table summarizes some of the recent works related to the use of chitosan-based delivery for the treatment of cancer (Table 1).

Chitosan NPs reach tumor sites through passive diffusion due to increased permeation and retention, effects commonly observed in solid tumors. Given its widespread application in both anticancer DDSs and gene delivery systems, chitosan has emerged as a promising delivery vehicle. Although it has proven to be effective in improving the bioavailability and release profile of several FDA approved anticancer drugs, there are currently no FDA-approved chitosan-based therapeutics for cancer diagnosis or treatment in the market [28]. Challenges associated with chitosan delivery persist, including factors such as surface charge density, scaling up, the grade and concentration of the polymer, pH sensitivity, and solubility. Consequently, there has been extensive research on surface-conjugated chitosan with targeting ligands for the overexpressed receptors on tumor, improving the overall safety and physicochemical profile of this drug delivery system [29,32,35].

#### 3.1.2. Hyaluronic Acid

**Figure 2:**
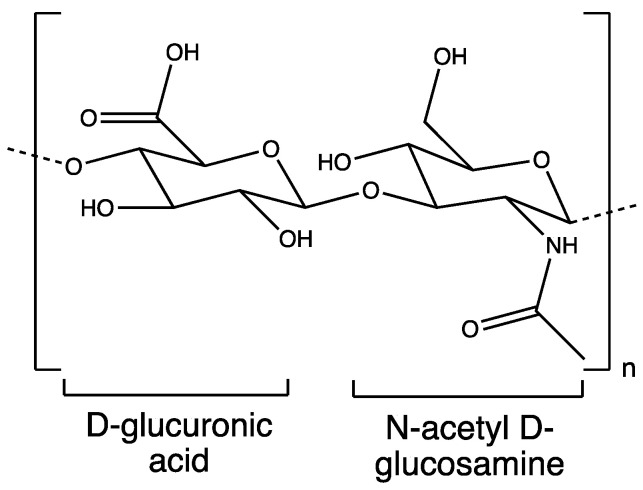
Chemical structure of HA.

Hyaluronic acid (HA), also known as hyaluronan, is ubiquitously present in all vertebrates, including humans, and it is particularly abundant in the synovial fluid and vitreous humor. As a component of extracellular systems, it is synthesized by HA synthase in plasma membranes [43]. Structurally, HA is an anionic linear polymer composed of a non-sulfated glycosaminoglycan chain with repeating disaccharide subunits of N-acetyl D-glucosamine and D-glucuronic acid (Figure 2) [44,45]. The molecular weight of HA spans a wide range, from 5 kDa to 20,000 kDa (low) or 1 MDa (high) [43,46]. The size and the concentration of the polymer vary depending on the source. LMW HA exhibits a good aqueous solubility, while polymers with weights exceeding 200 kDa possess an excellent water-holding capacity, playing a crucial role in maintaining hydration [44].

The molecular weight of the HA polymer plays a pivotal role in indicating its function in cancer progression [43,47]. LMW or oligo-HA is more prominently present in the periphery of the tumor cells and often correlates with a higher expression of CD44. CD44, a transmembrane glycoprotein, is typically overexpressed on cancer cells, exhibiting a strong affinity for HA, leading to cancer progression [48]. The binding of HA to its receptor leads to the aggressiveness of solid tumors, mainly by the migration and proliferation of tumor cells [44]. This uncontrolled growth can be migrated by externally administering HMW HA, which has a lower affinity for CD44 [49,50]. In addition to its affinity for overexpressed receptors, the bio-adhesive property of HA acts synergistically to facilitate cellular uptake [51]. Furthermore, as a negatively charged molecule, HA significantly prolongs the circulation half-life of the encapsulated therapeutics [52,53]. Reactive oxygen species (ROS) and enzymes like hyaluronidase-like protein and hyaluronidases degrade HA under strong acidic (pH < 4) or basic (pH > 11) conditions, facilitating the release of antitumor agents at the target site [43]. This may enhance the overall therapeutic efficacy of the antitumor therapeutics. HA NPs have gained popularity among polymer-based DDSs, serving as both standalone carriers and copolymers for targeting metal NP frameworks in the tumor microenvironment [48,51,54,55,56,57]. It also provides a unique platform for co-delivering genes and small xenobiotics, as well as the prodrug delivery of commonly used antitumor drugs to overcome chemo-resistance and improve the treatment efficacy [58,59,60,61]. Table 2 summarizes some of the recent applications of HA-based DDSs in cancer therapy.

HA-based DDSs has been successfully applied to evade the limitations of conventional cancer therapy. Although the anticancer formulations that have reached clinical trials have been limited in recent years, the in vivo efficacy studies of HA-based DDSs in animal models represent great success.

#### 3.1.3. Alginate

**Figure 3:**
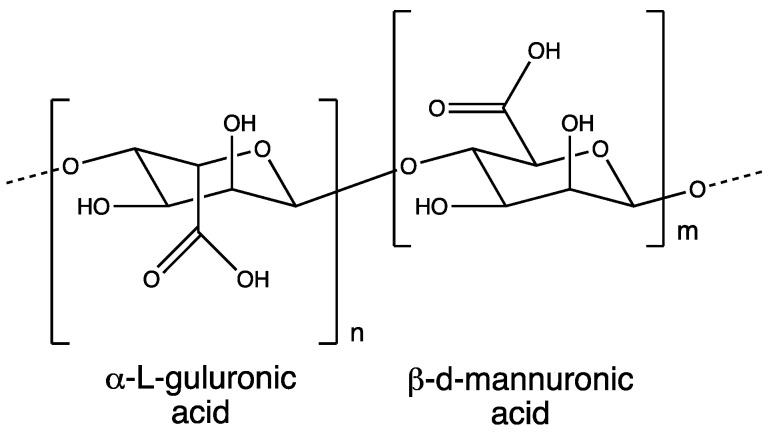
Chemical structure of alginate, consisting of repeating subunits of α-l-guluronic acid (G, n) and β-d-mannuronic acid (M, m).

Alginate belongs to the class of naturally occurring polysaccharides, widely available in nature and obtainable from various brown marine weed species, such as Ascophyllum nodosum, Laminaria digitate, Laminaria hyperborea, Laminaria japonica, Macrocystis pyrifera, and Ascophyllum nodosum. In addition to algae, certain microorganisms, like Azotobacter and Pseudomonas, can also serve as a source of alginate [67,68,69]. This linear polymer features irregularly repeating subunits of β-d-mannuronic acid (M, m) blocks and C-5 epimer α-l-guluronic acid (G, n) blocks, linked by β (1→4) linkages (Figure 3) [67,68,70,71]. Commercially, alginate is available in the form of sodium salt and alginic acid with a molecular weight ranging between 30,000 and 400,000 g/mol. Typically, alginate exhibits a good solubility at an aqueous neutral pH and has a significant water retention capacity [72]. However, its physicochemical properties vary considerably based on the repeating M and G subunits. Repetitive heterogenous subunits (MG and GM) are less likely to solubilize than homogenous subunits (MM and GG) at an acidic pH [72,73]. Therefore, the selection of an appropriate polymer grade is crucial for drug delivery. The ease of handling, cost-effectiveness, versatility, and biocompatibility make alginate a viable option for natural polymeric DDSs.

Alginate NPs can be synthesized through methods such as gelation/emulsification, complexation, solvent evaporation, precipitation, and cross-linking [71]. However, the use of cross-linking agents is less frequent due to their toxicity [67]. On the other hand, alginate hydrogels can be synthesized by gelation on interaction with divalent cations, such as barium and calcium. The gel contracts at an acidic pH and expands due to destabilization at a neutral or alkaline pH, facilitating drug release and making it a suitable drug delivery vehicle for orally administered drugs [71]. Chemotherapeutic compounds encapsulated within an alginate core can be released through diffusion or biodegradation other than the EPR effect [69]. Properties such as delayed or controlled release at the target tumor site, improved bioavailability, and the enhanced encapsulation of therapeutics have positioned alginate nano-systems as a promising option for cancer therapy (Table 3) [74].

Alginate NPs offer various advantages; however, the use of alginate as a nanomaterial is restricted due to the source-related variation in the molecular weight of the polymer [71,72]. Studies have also revealed that alginate hydrogels may experience leaching of heavy metals, initial burst release due to a leaky structure, and pH-dependent instability [71]. Researchers have successfully addressed some of these limitations by employing the subsequent cross-linking of alginate or by introducing copolymers to improve the mechanical strength of alginate-based delivery systems [75]. Extensive research concerning the safety profile of this carrier system is required.

#### 3.1.4. Cellulose

**Figure 4:**
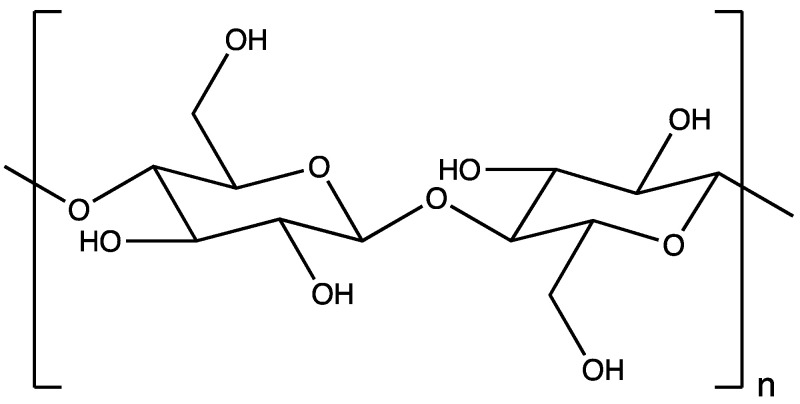
Chemical structure of cellulose.

Cellulose stands out the most abundant structural polymer derived from nature [81]. It exists as a linear polysaccharide, comprising hundreds to thousands monomer subunits of D-glucose linked together by β (1-4) glycosidic linkages (Figure 4) [81,82,83]. This renewable, cost-effective, and easily modifiable polymer, known for its excellent mechanical strength, is extensively utilized in the NP industry. The selection of the cellulose source (plants, tree bark, marine plants, algae, or bacteria) for NP production is crucial, as it can impact the size, morphology, and crystallinity of the resulting NPs [82,83,84]. For example, seaweed has a much higher turnover than land plants, while cellulose from cotton plants has more exposed active functional groups [85]. Additionally, the surface charge of cellulose carriers also depends on the extraction method employed, which may involve acidic hydrolysis (sulfuric acid, hydrochloric acid, maleic acid, or phosphoric acid), enzymatic hydrolysis (using cellulase and endoglucanase), oxidation (in the presence of NaIO_4_, hydrochloric acid, 2,2,6,6-tetramethyl-1-piperidine-N-oxy or ammonium persulfate), or mechanical refining (including processes like ultrasonication or homogenization). After extraction, the crude cellulose is referred to as cellulose nanocrystals or nanowhiskers. Commercially, it is available in a wide size range from a few hundred micrometers to 50 nm [84].

Cellulose nanocrystals or cellulose nanowhiskers possess a crystalline nature with pH sensitivity, mucoadhesive properties, gel-forming capabilities, and hydrophilic characteristics. The presence of inter- and intra-molecule hydrogen bonding makes cellulose insoluble in both aqueous and most organic solvents [86]. Despite solubility concerns, cellulose has been used to prepare highly stable NPs owing to its biodegradable, tasteless, odorless, and hydrophilic nature. The solubility of cellulose can be improved by chemically modifying the hydroxyl groups through processes such as esterification, etherification, carboxylation, and acetylation [83,84]. Some extensively researched cellulose derivatives include methylcellulose, carboxymethyl cellulose, and hydroxypropyl methylcellulose [87,88]. The preparation of NPs using modified cellulose follows similar methods as those for other natural polymers, including cross-linking, ionic gelation, and nanoprecipitation. Modified cellulose can be utilized alone or in combination with other organic or inorganic ligands to enhance the targeting of the delivery system [89]. Cellulose nanocarriers are well studied for various administration routes, such as oral, local, or topical. However, intravenous dosing is avoided due to its crystalline structure [90]. The sustained release, enhanced cellular interaction, improved absorption, and an overall reduction in the dose associated with cellulose NPs can address many adverse effects of chemotherapy (Table 4) [83].

Cellulose administration poses several challenges, including (1) immunogenic responses upon being identified as a foreign particle, (2) being cytotoxic to healthy host cells due to untargeted cellulose-based vehicles, and (3) toxicity because of limited degradation both in vitro and in vivo [96,97]. To address these challenges, it is often recommended to coat it with biocompatible polymers, such as gelatin, chitosan, and PLGA [84]. Furthermore, some in vivo studies have demonstrated DNA double-strand breaks and sperm abnormalities associated with extended pharyngeal exposure with cellulose-based products [84,98]. Hence, factors such as the choice of source, grade, and the method of cellulose preparation are crucial considerations when designing a cellulose-based DDSs.

#### 3.1.5. Gelatin

**Figure 5:**
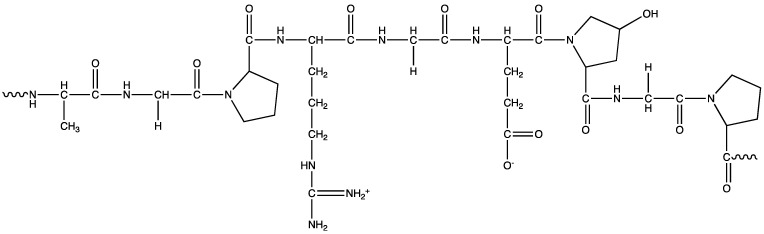
Chemical structure of gelatin.

Gelatin, derived from collagen, is a natural polymer recognized for its low toxicity, biocompatibility, and biodegradability. Approximately 30% of the key structural proteins in the human body consist of collagen and can be found in various forms, such as fibrous tissues, bones, blood vessels, and the GI tract (Figure 5) [99,100]. Bovine is the most common source of gelatin available for purchase, obtained through the partial acid or alkali hydrolysis of collagen, resulting in type A or type B gelatin, respectively. Gelatin is an amphiphilic macromolecule comprising positively charged (lysine and arginine), lipophilic (valine, leucine, isoleucine, alanine, etc.), and negatively charged (aspartic and glutamic acid) amino acid sequences, constituting ~35% of the structure [101,102]. The backbone of gelatin consists of repeated residues of proline, hydroxyproline, and glycine, resulting in a triple helical structure unique to collagen, and consequently, to gelatin. Due to its distinctive helical structure and the presence of numerous active groups, both anionic and cationic, gelatin serves as a stable and versatile carrier for delivering a wide array of therapeutic agents.

Gelatin has multifaceted roles in the biopharmaceutical industry, acting as a binder, stabilizer, gelling agent, or a hemostatic agent. Consequently, it has gained recognition as drug carrier for commonly administered hydrophilic and lipophilic anticancer drugs, including PTX, DOX, DTX, cisplatin, and gemcitabine [100]. The preparation of gelatin NPs involves techniques such as emulsification solvent evaporation, desolvation, coacervation-phase separation, and nanoprecipitation [102,103]. Gelatin NPs have various drug release mechanisms, either individually or in combination, including self-diffusion, bioerosion, and gelatin degradation by MMPs [100,104]. The literature supports the notion that gelatin NPs ensure drug encapsulation and result in sustained release, leading to a reduction in the overall dose [105]. Furthermore, gelatin has proven to be an effective non-viral vector, offering remarkable advantages in delivering genetic material, such as rapid uptake by cells, escape from endosomal degradation, and suitability for intravenous delivery [106,107,108].

Gelatin is recognized as a substrate for MMPs, which are overexpressed at the BBB and within the tumor environment. This characteristic provides an effective strategy for targeted therapy, particularly for brain cancers [109,110]. Gelatin NPs are versatile, facilitating interactions with various ligands (such as iron oxide for gene delivery, FA for cancer cells, etc.) for targeted cancer delivery [108]. Some of recent advancements in the field of gelatin-based DDSs are listed below (Table 5).

Gelatin NPs have effectively addressed numerous challenges associated with other polymeric NPs by serving as carriers for both hydrophobic and hydrophilic drugs. However, in comparison to other naturally occurring polymers, the synthesis of gelatin is a complex process, limiting its exploration in the field of DDSs [113]. The hydrolysis of gelatin can vary based on the source of collagen, including factors such as age the tissue type and animal species, making it challenging to maintain the overall nature and composition of the polymer [107]. Subsequently, they may be identified as foreign entities and cleared by the RES. Gelatin NPs are also known to develop a dynamic protein corona on their surface, potentially leading to toxic effects. Some of these limitations can be alleviated by modifying the surface of the NPs with PEG [102,112]. Optimizing the polymer synthesis process, size and zeta-potential, and the composition of NPs is crucial for advancing gelatin NPs towards market application.

#### 3.1.6. Dextran

**Figure 6:**
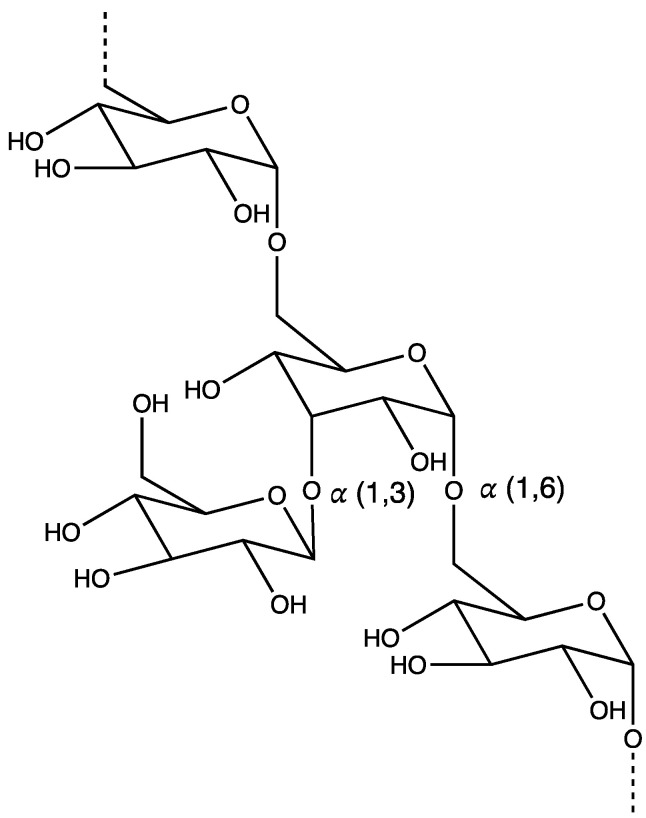
Chemical structure of dextran.

Dextran is a neutral polysaccharide obtained through the catalytic conversion of sucrose present in anaerobic bacterial species, such as Streptococcus and Leuconostoc. This branched and hydrophilic biopolymer consists of a linear chain of repeating glucose monomers joined by α-1,6 glycosidic linkages along with some branched glucose subunits, forming α-1,2, α-1,3, and α-1,4 glycosidic bonds (Figure 6) [114,115,116]. The bacteria are fermented or chemically synthesized for the production of dextran on a large scale. The molecular weight of dextran polymers can vary widely; however, for clinical use, they are available with 40, 60, and 70 kDa molecular weights [115,117]. Their high solubility (both aqueous and organic), non-immunogenicity, biodegradability, and biocompatibility allow for the efficient delivery of hydrophobic therapeutics using dextran-based carriers [115]. Dextran is very stable, thereby protecting the encapsulated moiety against enzymatic degradation in the stomach and small intestine. Consequently, its neutral charge facilitates absorption through the negatively charged intestinal wall, making it a suitable candidate for oral delivery [115]. Dextran serves as a substrate for dextranase residing in the large intestine, leading to enzymatic disintegration and biodegradation.

Similar to cellulose, dextran is readily modified via esterification, oxidation, and etherification, owing to the presence of numerous active hydroxyl groups. These modifications are necessary for transforming the highly ordered crystalline structure into an amorphous state, required for the construction of NPs [115]. Dextran-based carriers can be formulated through various techniques, such as prodrug approach, self-assembling NPs, nanogels, and micelles [118,119,120,121,122]. Although NPs do not disintegrate rapidly, as mentioned earlier, the ester bond in prodrug delivery systems is easily broken in the acidic tumor microenvironment, facilitating drug delivery at the targeted site. Dextran NPs can be formulated using microfluidics, nanoprecipitation, or emulsification. Dextran nanocarriers holds significant potential for mitigating the adverse effects associated with the high doses and drug resistance observed in chemotherapy by co-delivering small anticancer drugs with antibodies [123]. Additionally, they are considered superior in terms of drug encapsulation efficiency as compared to FDA-approved PLGA NPs [124]. Below are some recent works highlighting dextran-based DDSs (Table 6).

Delivering anticancer therapeutics via dextran derivatives is a relatively new concept and is gaining considerable attention in the field of nanocarriers [124]. However, several obstacles must be overcome before reaching the translational phase. As a biopolymer, maintaining source-to-source and batch-to-batch consistency is crucial. While the abundance of active groups is essential for polymer modifications, it may lead to undesirable interactions, resulting in altered physicochemical properties and thereby unexpected outcomes [127]. Therefore, extensive optimization and biosafety measurement are urgently needed for dextran-based DDSs. Overall, dextran represents a versatile nanocarrier that can take various forms, like nanogels or/and prodrugs, offering promising solutions to circumvent the common challenges associated with chemotherapy, including chronic treatment, poor aqueous solubility, and the instability of antitumor agents.

In summary, natural polymer-based carriers provide preventive measures along with their biocompatible, biodegradable, low toxicity, low immunogenicity, abundance, and renewability profile. They serve as an excellent platform for enhanced cellular uptake, controlled and sustained drug release, targeted delivery, customizability, and compatibility in combination therapy. These carriers have the potential to address many challenges associated with the current chemotherapy.

### 3.2. Synthetic Polymer-Based DDSs

Many synthetic polymers have been investigated as nanocarriers of DDSs for treating cancer. Due to their unique physicochemical properties, drugs can be encapsulated/conjugated by synthetic polymers with a high efficiency, to specifically target the cancer tissue and have extended drug release at the tumor sites. In recent decades, more insights have been obtained into smart nanoplatforms with specific chemical functionalities, for example, amphiphilic block copolymers, polymer-based prodrugs, stimuli-responsive polymers (pH sensitivity, ROS, GSH, thermo-responsive, enzymes, etc.), and fluorinated polymers. Moreover, synthetic polymers have attracted more attention than natural polymers because of the potential for the modifications of their structure and physicochemical properties. Synthetic polymers contain a broad capacity to encapsulate more bioactive molecules, not only small drugs, but also nucleic acids, peptides, and proteins [128]. Moreover, some synthetic polymers have improved stability due to the covalent cross-linking of the core via disulfide bonds [128]. Covalent cross-links can be cleaved under specific intracellular conditions, improving the endosomal escape after endocytosis, resulting in a more efficient drug delivery [128]. The synthetic polymers used in DDSs must be stable in the blood stream with a low toxicity and immunogenicity and need to protect the drug from degradation before reaching the target sites, where it has to remain active. Multiple PEGylated and PLGA nanoformulations have been successfully developed and approved, for example, Adagen^®^ (in the market in 1990, the first PEGylated protein) [129], Doxil^®^ (in the market in 1995, DOX in PEGylated liposomes) [130] and Movantk^®^ (in the market in 2014, PEGylated naloxone) [131], Onpattro^®^ (siRNA in PEGylated lipid nanoparticles in 2018) [132], and the most recent COVID-19 mRNA vaccines [133]. The following section is focused on synthetic polymer-based DDSs with specific chemical functionalities used in cancer therapy that can improve the physical–chemical properties and efficacy of APIs.

#### 3.2.1. Hydrophobic/Hydrophilic and Block Copolymers

The most commonly used hydrophobic polymers are PCL (poly(ε-caprolactone)), PLA, PGA, PLGA, and PPO (polypropylene oxide), while PEG, PEI (poly ethyleneimine), and HPMA are the most commonly used hydrophilic polymers. The FDA has approved some biodegradable synthetic polymers as drug delivery vehicles, like PLGA, PLA, and PEG. PLGA is a biodegradable copolymer ester of two α-hydroxyacid (lactic and glycolic acids) nanocarriers, which can improve the therapeutic efficacy and was approved by the U.S. FDA for long-acting release (LAR) [8]. PLGA has been widely used to encapsulate anticancer drugs with a good biodegradability, high bioavailability, and less systemic toxicity. It has been investigated to encapsulate a wide category of drugs due to its versatility in degradation properties, minimal systemic toxicity, commercial availability, and its ability to improve the pharmacokinetics of encapsulated drugs. PLGA nanocarriers can be prepared by various methods, such as emulsification-evaporation, nanoprecipitation, and the spray-drying method. Recently, scientists have shown great interest in the functionalization or surface modification of PLGA-NPs using various cancer cell-targeting ligands, like aptamers, FA, and antibodies, or decorated with other polymers, such as HA and chitosan, which not only can reduce the local and systemic toxicities but also improve the internalization of DDSs for efficient and targeted cancer therapy. Block copolymer micelles show great potential for the delivery of drugs/genes, with their most relevant feature being the formation of a distinct core-shell architecture. Kataoka conducted a comprehensive review of block copolymer micelles for drug delivery, emphasizing the significance of the unique core-shell structure that is driven by various forces, including hydrophobic interactions, electrostatic interactions, metal complexation, and the hydrogen bonding of the constituent block copolymers [134]. This architectural feature plays a crucial role in enhancing the delivery efficiency and for pharmaceutical applications.

Various anticancer drugs, including PTX, DTX, DOX, dexamethasone, and 5-FU, have been successfully encapsulated in PLGA NPs, and some of the formulations have been approved by the U.S. FDA for clinical purpose [135]. Recently, various macromolecules, such as peptides, genes, and human growth factors, have been also incorporated into PLGA NPs and effectively delivered to tumors tissue. PLGA NPs are also a suitable platform for the dual drug delivery of chemotherapeutics with other synergistic drugs that have been widely used for the effective targeting of various cancers. The most recent PLGA NPs for combination delivery are presented in Table 7. He et al. fabricated PLGA NPs combined with the saturated fatty acid palmitic acid (PA) and DOX by using the double emulsion solvent evaporation method and showed a reduction in tumor growth and metastasis [136]. Along with immunomodulatory properties, the tumoricidal activity of PA has been demonstrated in various cancer types, such as breast, colorectal, liver, and prostate cancers. Biomimetic hydroxymethyl phenylboronic acid-conjugated PLGA NPs, loaded with artesunate (AS) and chloroquine (CQ), were investigated for dual-targeting delivery to colorectal cancer [137]. The developed biomimetic PLGA NPs were effectively accumulated at the tumor tissues with the inhibition of the tumor cell growth [137]. Additionally, PLGA NPs can be radiolabeled as an effective technique for cancer imaging. In 2022, Ekinci et al. developed lamivudine (LAM)-loaded PLGA NPs (PLGA-NPs-LAM), which were further radiolabeled with radioactive technetium (99mTc) for lung cancer diagnosis [138]. The results demonstrated that the radiolabeling of PLGA-NPs-LAM with 99mTc effectively improved uptake by A-549 cells, suggesting it as a promising alternative agent for lung cancer imaging [138].

Although PLGA nanocarriers have distinctive properties, PLGA nanocarriers are limited because PLGA NPs can be easily recognized and rapidly cleared by the RES through liver [139]. PEG-conjugated PLGA polymers have been designed to improve the characteristics of these nanocarriers with a good immune tolerance. Moreover, PEGylated lipid-PLGA polymers can significantly improve the stability of NPs and further improve the drug cellular uptake, resulting in more in vivo therapeutic efficacy [140]. PEG is frequently used as the hydrophilic stealth polymeric for DDSs due to its excellent biodegradability and biocompatibility. Coating NPs with PEG provides many benefits, including the stabilization of formulations by reducing non-specific blood protein binding, preventing aggregation and macrophage clearance by the RES, and enhancing the blood circulation time [140]. Despite the potential benefits of PEG, it can also reduce the cellular uptake of the drug. Therefore, the degree of PEGylation must be carefully considered when developing PEG-based DDSs.

**Table 7:** Recent applications of PLGA-NPs in cancer therapy.

Delivery System	Drug	Methods	Inference	Ref.
HA-PLGA/PF68/PF127-NPs	Irinotecan (IRT)	Microfluidic-assisted nanoprecipitation process	Improved physicochemical behavior drug with high drug loading.	[141]
Dual receptor-targeted DTX-PLGA-NPs	DTX	Single emulsion solvent evaporation technique and further covalent conjugation	Micro- and nanosized carriers for imaging, chemotherapy, hyperthermia, and glioma.	[142]
PEG-coated PLGA-NPs	Curcumin	Solvent displacement method, PEGylation, and ligand conjugation	The ligands HA or FA conjugated with PLGA-PEG showed better in vitro efficacy and target ability.	[143]
CD56 antibody-conjugated PLGA-NPs	IRT and Stattic	Double emulsion solvent evaporation	Improved cellular uptake and in vitro efficacy and efficient active targeting of lung cancer cells.	[144]
CS-FA-PLGA-DTX	DTX	Nanoprecipitation approach	High drug-loading efficiency and controlled drug release, and high level of receptor-mediated internalization.	[145]
PLGA-NPs	Raloxifene hydrochloride (RAL)	Emulsion solvent diffusion evaporation	Improvement in stability at different temperatures and increase the in vitro efficacy at a lower concentration.	[146]
PLGA-NPs	Afatinib	Emulsification followed by solvent evaporation	Localized inhalational drug delivery for small lung cancer significantly improved cytotoxicity and cellular uptake.	[147]

#### 3.2.2. Dendrimers and Hyperbranched Polymers (HBPs)

Dendrimers and HBPs are gaining considerable attention as drug delivery vehicles for cancer therapeutics, owing to their unique three-dimensional structures with a hydrophobic core. Dendrimers are characterized by their nanosized, biocompatible, highly ordered, and mimic tree-branching macromolecule structure with a terminal function group [148]. They are classified based on their branching unit, with the central branch core dendrimer being known as generation 0 (G0) and the subsequent generations defined as G1, G2, G3, and so forth [149]. Dendrimers can be prepared by divergent or convergent methods, allowing for encapsulation and electrostatic or covalent conjugation with drugs [150]. Unlike dendrimers, other HBPs are cost-efficiently synthesized in a single step, resulting in irregular and highly branched macromolecules with multiple terminal functional groups [151]. In addition to drug delivery, dendrimers and HBPs have found widespread applications in various areas, including wastewater treatment, catalysis, and bioimaging.

Dendrimers readily form stable micellar or globular symmetric structures and can be easily surface-modified to enhance the water solubility of encapsulated drugs [152]. Dendrimers provide a higher stability than liposomes and micelles under various conditions, including high temperature, pressure, shear stress, and dilutions, thereby improving pharmacokinetic factors [153]. They can be chemically modified for both active and passive targeting. PEGylated dendrimers contribute to the protection against proteolytic degradation and the extension of the circulation half-life of loaded chemotherapeutics [154]. Notable examples such as polypropylene imine (PPI) and polyamidoamine (PAMAM) dendrimers have shown success in biomedical applications [152]. Additionally, PEI and polyglycerol (PG) dendrimers exhibit a pH-dependent release pattern, favoring a faster release at pH 5-6 compared to pH 7, making them suitable for targeting anticancer drugs within the TME [155,156]. Moreover, dendrimers show promise in overcoming challenges in gene delivery, particularly for large hydrophilic molecules prone to enzymatic degradation with limited cell membrane penetration [156,157]. Dendrimers also play a significant role in the combined delivery of multiple chemotherapeutics, such as siRNA, to mitigate nonspecific toxicity and enhance specificity by surface functionalization with targeting ligands [158]. Recent studies have demonstrated the efficacy of FA-conjugated polyamidoamine dendrimer-based NPs for the co-delivery of siRNA and cis-diamminodichloride platinum (CDDP) in a dual-targeting antitumor therapy for lung cancer [159]. Additionally, the conjugation of mAbs with drug-loaded dendrimers improves the binding specificity at overexpressed receptors in cancer cells, enhancing efficacy at the target site compared to the free drugs [160]. For example, the conjugation of recombinant humanized mAbs trastuzumab with PAMAM dendrimers improved the efficacy and targetability of the encapsulated drugs (DTX or PTX) in the treatment of HER-2-positive breast cancer [161,162]. HBPs synthesized by Pearce et al. were utilized for the targeted delivery of DOX to PSMA-overexpressed prostate cancer, demonstrating improved therapeutic effects [163]. Table 8 provides an overview of the recent applications of dendrimers and HBPs as drug carriers for the single or co-delivery of drugs.

#### 3.2.3. pH-Responsive Polymers

The weakly acidic microenvironment (pH < 6.5) around the tumor tissue is caused by large number of acid metabolites generated through glycolysis in tumor cells. pH-responsive polymer-based delivery systems allow the selectively and targeted release of chemotherapeutics at the tumor tissue, and then improve the in vivo efficacy. Additionally, these pH-responsive polymers are relatively stable in normal tissues, and significant changes occur in their chemical structures and the physical state after being exposed to the acidic pH environment mainly at the tumor site and in lysosomes, which leads to the rapid changes in the nanomaterials and drug release [170].

pH-Responsive polymers can be broadly classified into two groups: polymers that have ionizable groups and polymers that contain acid-labile linkages [80]. In general, polymers with ionizable moieties are either basic polymers that accept protons at a relatively low pH or acidic polymers that release protons at a relatively high pH. Classically, polymers with basic groups include amines, pyridines, morpholines, piperazines, pyrrolidine, imidazole such as poly (2-(dimethylamino) ethyl methacrylate) (PDMAEMA), poly(N-acryloylmorpholine) (PNAM), poly(N-vinyl imidazole), and dendrimers, while common polymers with acidic moieties include sulfonic, carboxyl, phosphoric, and boronic acids groups like poly(methacrylic acid) (PMAA), poly(vinyl-phosphonic acid) (PVPA), PGA, poly(vinyl-sulfonic acid) (PVSA), and poly(aspartic acid) (PASA), which can be protonated or deprotonated at different pH values [80].

Another category of pH-responsive polymers usually contains acid-labile covalent linkages, which are prone to cleavage, leading to the dissociation of polymer aggregates or the degradation of polymer chains at altered pH conditions. Polymers with acid-labile linkages exhibit slower internal structural transitions due to covalent bonding, which facilitate their applications in DDSs. Recently, Nour et al. developed a pH-stimuli-responsive poly(ethylene glycol/acrylic acid) (PEG/PAA) nanogel and further evaluated it in vitro against four human cancer cell lines HepG2, A549, MCF-7, and HCT-116 [171]. Guo et al. fabricated self-assembled pH-responsive NPs loaded with a TMEM16A inhibitor for the dual-targeting antitumor therapy for lung adenocarcinoma, which demonstrated a significant antitumor activity with dual-targeting therapy using a xenograft mouse model [172]. Additionally, these polymers are also applicable for the imaging of a wide range of tumors [173]. The developed system simply accesses the acidic tumor microenvironment or is internalized into endocytic organelles within the tumor endothelial cells, demonstrating a broad tumor specificity across a variety of tumor models [174]. Table 9 summarized some of the recent applications of pH-responsive polymer-based DDSs in cancer therapy.

#### 3.2.4. Redox-Responsive Polymers

Redox-responsive polymers represent another example of tumor-targeted drug delivery systems due to endogenous stimuli, which can target biorelevant redox molecules and undergo structural changes to release payloads, including glutathione (GSH), ROS, and hydrogen sulfide (H_2_S) [181]. The presence of a low concentration of reduced GSH (2 to 10 μM) in the extracellular space and a high concentration of GSH (2 to 10 mM) in the intracellular environment creates a redox potential difference across the cell membrane. This forms a basis for delivering therapeutics encapsulated in ROS/reduction-responsive polymers [182]. Additionally, the TME has a significantly higher GSH concentration than normal tissues, resulting in the intracellular delivery and release of the therapeutics at the tumor site [183].

An ideal redox-responsive polymer should contain redox-responsive subunits that are stable in the oxidizing extracellular space but are reduced in the intracellular environment. One of the most studied GSH-responsive polymer modifications is the incorporation of disulfide bonds in either the polymer backbone, polymeric side chains, or cross-linked micellar-based carriers [184]. This can be achieved by either self-assembly, cross-linking, or by conjugating the drug with the polymer of interest. In the oxidizing media, disulfide bonds remain stable; however, they form thiol groups in a reducing condition, compromising the integrity of the micelles and ultimately releasing the encapsulated drug. Other commonly used ROS-responsive polymer units are thioketal, arylboronic acid/ester, and succinimide-sulfide bonds [185]. The diselenide group is sensitive to both oxidative and reducing conditions and can be used as a dual redox-responsive polymer. Redox-responsive polymers have been widely studied in the literature and some of the examples are listed in Table 10.

Overall, redox-responsive polymers have the ability to prevent the initial burst release of the drug and can prolong the circulation half-life, thereby improving the therapeutic efficacy of the small therapeutics. Despite the controlled and targeted delivery nature of redox polymers, limitations like premature or non-specific release of the drug, low drug-loading capacity, and low tumor accumulation are associated with these micellar formulations and limit their extensive use [185].

#### 3.2.5. Other Stimuli-Responsive (Thermo-, Hypoxia-, and Enzyme-Responsive) Polymers

In recent years, there has been a notable growth in the development of stimuli-responsive polymer-based DDSs characterized by sustained and controlled drug release properties. Thermo-responsive, hypoxia-responsive, and enzyme-responsive polymers are key players in this field and have attracted significant attention. Stimuli-responsive DDSs hold promising potential for improving cancer treatment efficacy by minimizing off-target effects and enhancing drug bioavailability.

Temperature has been extensively explored as a promising candidate in stimuli-responsive polymer systems, primarily due to its ease of modulation and applicability in drug delivery applications. Thermo-responsive polymers undergo a phase transition from hydrophilic to hydrophobic within a specific temperature range, known as the lower critical solution temperature (LCST). Poly (N-isopropylacrylamide) (PNIPAAm) stands out as the most extensively studied synthetic thermo-responsive polymer, owing to its sol-gel phase transition property. The LCST of PNIPAAm is near 32 °C, and PNIPAAm exhibits a hydrophilic coil below the LCST, while above 32 °C, PNIPAAm chains sharply collapse into a hydrophobic globule [194]. Another noteworthy thermo-responsive polymer is Pluronic F127, recognized for its in situ gel formation capacity at the body temperature of 37 °C [195]. Innovative polymerization techniques, such as click chemistry reactions and controlled radical polymerization, offer opportunities to create materials with specific responsive behaviors. An illustrative example is the work by Chen et al., who developed a thermo-responsive polymeric dexamethasone (Dex) prodrug (ProGel-Dex) based on HPMA for treating osteoarthritis (OA). This prodrug, when in an aqueous solution, remains a free-flowing liquid at 4–20 °C, but is transformed into a hydrogel when exposed to temperatures above 25 °C [196]. This feature facilitates the administration and sustained retention of the Dex prodrug, providing complete and prolonged relief from pain and inflammation in the context of OA.

Hypoxia constitutes a critical component of the TME due to inadequate vascular oxygen supply, playing an important role in cancer progression and therapeutic efficacy. Normal tissues maintain oxygen tensions in the range of ~30–70 mmHg; however, the oxygen tension in most tumors ranges from 2.5 mmHg to 7.5 mmHg [197]. Consequently, the development of hypoxia-responsive systems has emerged as an innovative hotspot in cancer therapeutic delivery. Hypoxia-responsive carriers exhibit the ability to undergo structural changes in response to hypoxic conditions, facilitating the release of therapeutic agents within the TME. For instance, Mamnoon et al. developed estradiol-conjugated hypoxia-responsive DDSs loaded with DOX for targeted drug delivery to hypoxic niches in estrogen receptor (ER)-positive breast cancer, being the first report of targeted ER-mediated hypoxia-responsive polymeric drug carriers for cancer treatment [198]. Both hypoxia-activated DDSs and redox-responsive nanocarriers significantly impact the targeting of tumor hypoxia in cancer. Zhang et al. engineered dual-responsive nanocarriers (^DA^NP_CT_) encapsulating the photosensitizer chlorin e6 (Ce6) and hypoxia-activated prodrug tirapazamine (TPZ) to enable efficient PDT-boosted hypoxia-activated chemotherapy [199]. Notably, Zhao et al. investigated hypoxia-responsive polymeric nanoprodrug (PNPs) for the co-delivery of the photosensitizer 5,10,5,20-tetrakis(4-aminophenyl)-porphine (TAPP) and chlorambucil (CB) to enhance the overall therapeutic efficacy [200]. The photosensitizer TAPP converted oxygen to produce single oxygen (^1^O_2_) for PDT, creating a PDT-reduced hypoxia environment and subsequently improving the release of activated CB for effective cancer treatment [200].

The development of enzyme-responsive DDSs constructed from biodegradable polymers sensitive to specific enzymes represents a highly promising avenue within smart stimuli-responsive systems. The breakdown of the polymer network triggered by endogenous enzymes, leading to the release of the drug at the specific target sites, serves as the foundation for the design of enzyme-responsive DDSs [201]. The predominant approach for the enzymatic fabrication of polymeric nanocarriers involves self-assembly and polymerization methods. Enzymes such as elastase, trans-glutaminase (TGlu), horseradish peroxidase (HPR), MMPs, cathepsins, hyaluronidases, and tyrosinase (Tyr) are commonly employed in the preparation of these nanocarriers [202]. For instance, it has been reported that the prostate-specific membrane antigen (PSMA, also known as glutamate carboxypeptidase 2) level is much higher in prostatic cancer cells (100–1000-fold higher than normal prostate epithelial cells) [203]. Enzyme-responsive nanocarriers often incorporate amino acids as a side chain that can be covalently connected by the enzyme (TGlu-sensitive systems). Enzymes offer numerous advantages as triggers, including high chemical selectivity and substrate specificity, and they operate under mild conditions, such as a ~neutral pH, aqueous media, and low temperature [204]. Table 11 provides some of the recent applications based on stimuli-responsive polymers for cancer therapeutics.

#### 3.2.6. Targeting Ligands of Polymers

Polymers incorporating targeting ligands play an important role in the delivery of cancer therapeutics. These targeting ligands, which commonly include antibody fragments or mAbs, peptides, aptamers, and certain small molecules, are designed to selectively bind to receptors overexpressed in the cancer cell membrane. The conjugation of drugs and specific polymers, often through linkers that act as breakage points, results in the formation of PDCs or prodrugs, enabling the release of drugs at the targeted sites. A recent comprehensive review about PDCs for targeted cancer therapy can be found in [217]. The surface of nanocarriers can be further modified with various ligands, such as iRGD (arginine-glycine-aspartate peptides), enhancing the ability to actively target tumors [218]. Research efforts have extensively explored the identification of biomarkers overexpressed in tumor cells, including cell adhesion molecules, CD44, folate receptors (FR), transferrin (Tf), glucose transporter protein type 1 (GLUT1), LAT1, and biotin receptors, to refine the targeting strategies for cancer therapeutic delivery. Jin et al. developed a FR-mediated dual-targeting DDS to enhance tumor-killing efficiency and limit side effects [219]. The FR-mediated cathepsin B-sensitive DDS (FA-GFLG-SN38) comprised the FR ligand FA, the tetrapeptide substrate for cathepsin B (GFLG), and anticancer drug SN38. The results indicated that this novel delivery system effectively inhibited tumor proliferation [219]. HER2, an internalization-resistant receptor, overexpressed in cancer, serves as another attractive target. However, most chemotherapeutics target intracellular mechanisms, posing a significant challenge in exploiting HER2 overexpression to enhance targeting and anticancer efficacy. Radford et al. developed an HPMA copolymer-based DDS by conjugating a small (7 kDa) HER2-binding affibody peptide, demonstrating a significant increase in intracellular delivery compared to the unconjugated HPMA polymer [220].

The GLUT1, LAT1, and ASCT2 pathways have been investigated as targets for gliomas and hepatocarcinoma, driving the development of various polymer-based strategies to enhance brain delivery [221]. GLUT1 is expressed in the endothelial cells of the BBB, facilitating the transport of glucose and DHA into the brain [222]. Due to the high affinity of D-glucosamine and GLUT1, D-glucosamine-based DDSs showed enhanced tumor uptake and increased tumor distribution through GLUT1-mediated endocytosis [221]. A poly-prodrug-based micelle formulation was developed by conjugating PTX with PEG-Azo via a disulfide link (Glu-PEG-Azo-IR808-S-S-PTX) for mitochondria-specific drug delivery and tumor thermal therapy. Such nanocarriers effectively destroyed the mitochondria and down-regulated ATP production [223]. LAT1, an amino acid transporter overexpressed in BBB, glioma cells, and other tumors, has been a focal point of polymer-based prodrugs utilizing LAT1-mediated transport [224]. These LAT1-targeted prodrugs primarily rely on the large hydrophobic amino acids L-Phe and L-Tyr [225]. Moreover, polymers with methionine (Met)- or cysteine (Cys)-like structures on the side chains exhibited a LAT1-targeting ability, offering potential designs for LAT1-targeted DDSs [226]. ASCT2, another amino acid transporter overexpressed in various carcinomas, has been targeted using functionalized polymers through glutamine [227]. Glutamine-conjugated β-cyclodextrin loaded with DOX demonstrated specific accumulation in ASCT2-expressig breast cancer cell lines [228]. The uptake of DOX was significantly attenuated by L-γ-glutamyl-p-nitroanilide, a specific inhibitor of ASCT2. Table 12 provides an overview of the recent applications based on targeted polymer-based DDSs in cancer therapy.

#### 3.2.7. Fluorinated Polymers

Fluorine chemistry has emerged as a pivotal player in the field of pharmaceutics as a “arsine star”. Importantly, the continued investigation of fluorinated polymer bases for drug delivery is essential for further advancements in the field. The unique properties of fluorine and its derivatives find application in drug delivery to enhance metabolic stability, facilitate membrane permeation, and increase binding affinity. This approach has become paradigmatic in medicinal chemistry [236]. Notably, over 25% of marketed drugs contain fluorine atoms and approximately 20% of marketed drugs feature fluorine-containing motifs [237]. In 2018, 17 FDA-approved drugs containing fluorine were used to treat cancer, HIV and malarial and smallpox infections [238].

The integration of fluorine into polymers has demonstrated significant benefits in delivering APIs. Fluorinated polymers, known as fluoropolymers, exhibit both hydrophobicity and hydrophilicity due to their low surface energy and refractive index [236]. Recognized for their chemical and biological inertness, fluoropolymers exhibit low cytotoxicity by reducing interactions with serum components, cells, and tissues [239]. The introduction of fluorine atoms into polymers enhances transmembrane ability by reducing their basicity and cationic charge [239]. However, fluoropolymers are not available from natural sources, and fluorinated materials can be synthesized through various approaches, including free radical polymerization mechanism, emulsion polymerization, and polymerization in supercritical fluids [240]. The fluoroalkyl chains contribute to complex stability, resisting interference from phospholipids or proteins, ensuring stability in water and phospholipid phases. Recent studies have explored the use of fluoropolymers for the delivery of cancer therapeutics. For instance, Yang et al. developed a ROS-responsive fluorinated PEI vector for co-delivering plasmids in cancer therapy, providing a safe and high-performance platform for enhanced anticancer efficacy [241,242]. F-PEI and F-chitosan were also utilized for the transmucosal delivery of active peptides for the intravesical instillation therapy of bladder cancer [243]. Another innovative strategy involved fabricating personalized nanovaccines based on fluoropolymers for cancer immunotherapy, resulting in a robust immune memory against tumor rechallenge in an orthotopic tumor model [244]. Fluoropolymers combined with perfluorocarbon (PFC) can relieve tumor hypoxia and enable synergistic sonodynamic immunotherapy. Yang et al. developed PFC-loaded fluoropolymers (PFCE@THPPpf) as multifunctional nano-sonosensitizers [245]. Table 13 provides an overview of the recent applications of fluorinated polymers in cancer therapy.

## 4. Conclusions and Future Perspectives

In recent decades, polymer-based DDSs have received increased attention owing to their biocompatibility, enhanced bioavailability, and the ability to enhance the tumor accumulation and anticancer efficacy of novel therapeutics. These systems offer notable advantages, including the controlled release of cargos, targeted delivery to tumor sites, the reduction in side effects, prolonged blood circulation time, enhanced cellular uptake, and increased therapeutic efficacy, while minimizing damage to healthy tissues and off-target sites. The unique chemical structure of polymers allows for the conjugation of active-targeting moieties, offering the potential to improve pharmacokinetic properties. The integration of nanotechnology and advanced imaging techniques holds promise for further improving the precision and effectiveness of polymer-based DDSs in cancer treatment. Beyond cancer, the appeal of polymer-based DDSs extends to other diseases, such as inflammation-driven diseases, infectious diseases, arthritis, fibrosis, and more. While significant progress has been made in achieving sufficient drug concentrations at tumor sites and targeted drug delivery through the incorporation of multiple functionalities and moieties in additive models, many of these approaches fall short of adequately addressing biological barriers, thus limiting clinical utility. Despite these challenges, the future of polymer-based DDSs appears promising, offering the potential for more effective and less toxic therapies across various medical applications.

## Figures and Tables

**Table 1:** Recent applications of chitosan as a DDS in cancer therapy.

Delivery System	Drug	Methods	Inference	Ref.
Chitosan hydrogel	Dopamine-conjugated perylene	Covalent cross-linking	Biocompatible, elastic, and photosensitive gel significantly enhanced the phototoxicity of perylene compared to the free drug.	[36]
Nitrosalicylaldehyde; Aldehyde HA; Chitosan hydrogel	DOX and cisplatin	Covalent cross-linking	Sustained release of the drugs from the hydrogel at a physiological and slightly acidic pH value demonstrated antiproliferative effects and biodegradable properties.	[37]
Sialic acid; Cetuximab; Chitosan NPs	Gemcitabine	Ionic gelation	Increased bioavailability and reduced clearance along with enhanced antiproliferative activity and cell internalization of the targeted chitosan NPs.	[38]
HA dialdehyde; Chitosan NPs	siRNA	Ionic gelation	Targeted accumulation, inhibiting tumor growth by silencing the oncogene, and good blood compatibility.	[39]
T7 peptide; Carboxymethyl chitosan NPs	Docetaxel (DTX) and curcumin	Ionic gelation	Enhanced in vitro and in vivo antitumor effects compared to monotherapy and good biosafety.	[40]
Zein; Chitosan NPs	Curcumin and berberine	Anti-solvent precipitation method	Biocompatible, redispersible, and stable NPs demonstrated improved cytotoxicity, cell internalization, and apoptosis with anti-inflammatory properties.	[41]
PLGA; Chitosan NPs	DOX	Anti-solvent precipitation method	Sustained pharmacodynamics of DOX.	[42]

**Table 2:** Recent applications of HA as a DDS in cancer therapy.

Delivery System	Drug	Methods	Inference	Ref.
Fucoidan, Zein, HA NPs	Fisetin	Anti-solvent precipitation	Targeted delivery with significantly higher cytotoxicity.	[62]
L-glutamate, HA NPs	Gefitinib and vorinostat	Nanoprecipitation	Targeted therapy, reduced systemic toxicity, and substantial tumor growth inhibition.	[63]
HA NPs	Indocyanine green (ICG)	Self-assembly	Accumulation at the target site compared to healthy cells.	[64]
PEG HA NPs	Mitoxantrone	Anti-solvent precipitation	Significantly higher cytotoxicity to CD44-positive cells and apoptosis.	[65]
Alendronate sodium; HA NPs	Methotrexate	Self-assembly	Reduced off-target effects and improved antitumor activity.	[66]
β-cyclodextrin; Poly-l-lysine; HA NPs	DOX and oligo-RNA	Self-assembly and anti-solvent precipitation	CD44-mediated delivery of therapeutics and accumulation in tumors.	[61]

**Table 3:** Recent applications of alginate NPs in cancer therapy.

Delivery System	Drug	Methods	Inference	Ref.
Alginate microbeads	DOX	Microencapsulation	Reduction in the initial burst release and increase in the encapsulation efficiency.	[75]
Alginate NPs	Quercetin (leukemia)	Cold precipitation	Long shelf-life, high drug entrapment, sustained release, and improved cytotoxicity.	[76]
Chitosan; Alginate NPs	DOX	Ionic gelation	Sustained release and improved cytotoxicity.	[77]
Ferrous oxide, Gelatin, Oxidized alginate hydrogel	DOX	Physical mixing	pH-dependent release profile, and higher encapsulation efficiency.	[78]
Boronated chitosan; Sodium alginate NPs	PTX	Ionotropic gelation	Conjugation improved the mucoadhesive properties, very high encapsulation and drug-loading capacity, and sustained release.	[79]
Folic acid; Sodium alginate NPs	Diferourylmethane	Emulsion solvent evaporation method	Folic acid (FA) conjugation allowed sustained release and a higher cellular uptake.	[80]

**Table 4:** Recent applications of cellulose-based DDSs in cancer therapy.

Delivery System	Drug	Methods	Inference	Ref.
Carboxymethyl cellulose conjugation	Curcumin	Cross-linking	Enhanced permeation and anti-proliferation.	[91]
Chitosan; Ferrous oxide; Cellulose nanowhiskers	5-Fluorouracil (5-FU)	Co-precipitation and ionic gelation	pH-dependent release, higher anticancer effect, and low cost.	[92]
Cellulose nanocrystals	Curcumin	Nanoprecipitation	Sustained drug release, biocompatible, and improved cytotoxicity.	[93]
Chitosan; Disulfide cross-linked carboxymethyl cellulose NPs	5-FU and polypyrrole	Emulsification	Drug release upon acidic and redox stimuli, improved cellular uptake, and synergistic tumor growth inhibition.	[94]
Sodium carboxymethyl cellulose hydrogel	DOX	Cross-linking	Improved drug loading, controlled and prolonged release, and biocompatiblity.	[95]

**Table 5:** Recent applications of gelatin-based DDSs in cancer therapy.

Delivery System	Drug	Methods	Inference	Ref.
Iron oxide; Gelatin NPs	siRNA	Desolvation and cross-linking	Improved shelf-life, encapsulation efficiency, cytocompatibility, and anticancer activity.	[108]
Melanin; Gelatin NPs	Photoacoustic tumor imaging	Desolvation	Tumor-targeted accumulation, biocompatiblity, and substrate MMP degradation.	[110]
Oleic acid; Gelatin NPs	Sesamol	Desolvation and cross-linking	Significantly higher permeation after transdermal delivery and reduced IC50.	[111]
Concanavalin A; Gelatin NPs	Cisplatin	Desolvation and cross-linking	On-demand release system, targeted released of the drug due to interaction with MMPs, biocompatibility, and improved endocytosis.	[104]
PEGylated gelatin NPs	DOX; Betanin	Desolvation	pH-responsive controlled drug release, improved cellular uptake, cytotoxicity, and apoptosis.	[112]

**Table 6:** Recent applications of dextran-based DDSs in cancer therapy.

Delivery System	Drug	Methods	Inference	Ref.
Oxidized dextran NPs	DOX and CD147	Prodrug and self-assembly	Sustained and acid-sensitive release of the drug, prolonged blood half-life, and significant tumor growth inhibition.	[123]
Aldehyded dextran nanogel	DOX	Inverse microemulsion	Uptake by tumor cells and pH-sensitive drug release.	[119]
Deoxycholic acid; Dextran NPs	Silybin and PTX	Self-assembly	Passive targeting, tumor accumulation, and tumor growth inhibition.	[118]
Aldehyded dextran carrier	DOX	Prodrug conjugation	Internalization of particles, delayed drug release, and substantially high tumor penetration.	[125]
Lithocholic acid; Carboxymethyl dextran NPs	DOX	Self-assembly	Accumulation at the tumor site, rapid release of the drug in the reductive tumor environment, and extremely low release at a physiological pH.	[126]
Ethoxy acetalated dextran NPs	BRP-187	Microfluidics, emulsification, and nanoprecipitation	Enhanced encapsulation efficiency, and nanoprecipitation was the method of choice.	[124]

**Table 8:** Recent applications of dendrimer- and HBP-based DDSs in cancer therapy.

Delivery System	Drug	Methods	Inference	Ref.
Mitochondrial-targeted PAMAM dendrimers	Curcumin	Chemical conjugation	Selectively induced potent apoptosis and cell cycle arrest at G2/M with improved solubility.	[164]
FA-conjugated PAMAM dendrimers	siRNA and CDDP	Covalent conjugation to G4 dendrimer using PEI and PEG	Improved the therapeutic effects of HuR siRNA and CDDP against H1299 lung cancer cells.	[159]
Trastuzumab-conjugated PAMAM dendrimers	DOX and mAb	PAMAM and DOX were conjugated by using cis-Aconitic anhydride (CAA)	High toxicity of PAMAM-DOX-trastuzumab conjugates against HER-2-positive (SKBR-3) and -negative (MCF-7) breast cancer cells.	[165]
PG-co-PCL dendritic nano-structure	Gemcitabine	Copolymerization of the monomer mixture composed of glycidol and ε-caprolactone	Improved pH-dependent release with a better toxicity for both non-covalent- and covalent-conjugated gemcitabine against pancreatic cancer.	[166]
GSH-triggered HBP-based micelles	Camptothecin	Self-condensing vinyl polymerization strategy via the atom transfer radical polymerization (ATRP) of drug-contained monomers and hydrophilic macromolecular monomers	Superior stability with a high release and improved tumor cell growth inhibition.	[167]
Cy3-labeled G4 (G4-Cy3) and Cy5-labeled G6 (PAMAM) dendrimers (G6-Cy5)	Fluorescent dye Cy3 and Cy5	Surface-modified into amine-terminated bifunctional dendrimers	G6 dendrimer demonstrated a high delivery efficacy compared to G4.	[168,169]
Stimuli-eesponsive dendritic polymer-based nanococktail	Gefitinib and YAP-siRNA	Chemical conjugation and electric condensation	Induced tumor cell apoptosis through PDT and improved antitumor efficacy I cell line-derived xenograft and patient-derived xenograft tumor models.	[169]

**Table 9:** Recent applications of pH-responsive polymer-based DDSs in cancer therapy.

Delivery System	Drug	Methods	Inference	Ref.
pH-Responsive triple-sensitive nanogel	DOX	Sensitive monomer grafted onto sodium alginate	Targeted and controlled release of DOX in vitro.	[175]
pH-Responsive cross-linked chitosan/laponite RD NPs	DOX and Sorafenib (SF)	Cross-linking	pH stimulated the simultaneous in vitro release of DOX and SF; higher cytotoxicity against breast cancer cell lines.	[176]
pH-Responsive poly (MAA-co-IA) NPs	DOX and Methotrexate	One-pot biphase stratification approach	Improved tumor inhibition compared to plan DOX and methotrexate.	[177]
pH-Sensitive NPs	DTX	DTX and dihydroartemisinin conjugated with 4-arm-PEG via a hydrazone bond	Significantly increased the apoptosis of 4T1 cells and inhibited lung metastasis due to a synergistic effect.	[178]
pH-Sensitive NPs	5-FU and Leucovorin	Double emulsion and solvent evaporation	Showed a pH-responsive drug release and exhibited a significantly higher cytotoxic action.	[179]
pH-Sensitive NPs	DTX and Disulfiram	Nanoprecipitation method using microfluidics	Increased in vivo antitumor efficacy against a mouse orthotopic breast cancer model, while decreasing P-gp expression and preventing lung metastasis.	[180]

**Table 10:** Recent applications of redox-responsive polymer-based DDSs in cancer therapy.

Delivery System	Drug	Methods	Inference	Ref.
ROS-responsive dextran-based Pt nanoprodrug (PDPN)	DOX	One-pot chemical coupling of carbonylated methoxy PEG, dextran, and the cross-linking agent cisPt	PDPN-DOX displayed the reduction-responsive release of DOX and Pt with synergistic anticancer effects.	[186]
ROS-responsive and active-targeting drug delivery systems (AG-PEG-SS-PCL)	SF	Thin-film hydration method	Excellent antitumor effects and better tolerance.	[187]
Acrylamide-based NPs containing ROS-sensitive cross-linkers	microRNA	Electrostatic interaction between positively charged NPs and negatively charged microRNA	Stable dispersions were formed in biological media and enhanced microRNA release in the presence of GSH.	[188]
Thermo- and reduction-responsive copolymers	CPT	Self-assembly	GSH could trigger the release of CPT drugs and was promoted by NIR light-induced photothermal therapy.	[189]
pH/Reduction dual-responsive HA prodrug	Podophyllotoxin (PPT)	Self-assembly	Due to HA receptor-mediated endocytosis, HA-S-S-PPT accumulated at the tumor site and achieved excellent antitumor effects.	[190]
Reduction-responsive chemo-capsule-based prodrug	10-hydroxy camptothecin and DOX	Two-in-one cross-linking strategy to prepare the stimuli-responsive prodrug by virtue of delivery chemotherapeutics	Drug released from the prodrug in response to the reduction in the tumor microenvironment, enhancing tumor growth inhibition.	[191]
Reduction-responsive PEG nanodrug	PTX	Self-assembly	Nanodrug was selectively disassociated in the intratumor reduction microenvironment via the reduction of disulfide bonds to release PTX, and excellent in vivo antitumor efficacy while avoiding side effects was observed.	[192]
Chitosan-lipoic acid reduction-responsive (CS-LANPs)	Curcumin	Self-assembly	Increased tumor accumulation with a better tumor inhibitory activity in vitro.	[193]

**Table 11:** Recent applications of stimuli-responsive polymer-based DDSs in cancer therapy.

Delivery System	Drug	Methods	Inference	Ref.
Thermo- and pH-responsive copolymer hydrogels	Curcumin	Radical polymerization and swelling diffusion	Both temperature- and pH-responsive behavior with good biocompatibility.	[205]
Chitosan thermo-sensitive hydrogel	Gemcitabine hydrochloride, levofloxacin, 5-FU	Self-assembling	Precisely regulated the gelling time with potential for drug delivery and chemotherapy.	[206]
PNIPAM-b-PAzoMA	Iron oxide	RAFT radical polymerization	Shown excellent thermo-sensitivity and photosensitivity.	[207]
Thermo-responsive copolymer	siRNA	Chemical conjugation	The LCST of siRNA-conjugated thermo-copolymer was 38 °C with excellent cellular uptake and gene silencing.	[208]
Hypoxia-responsive polymer micelles (AA/ASP-AZO-Fc, AAAF)	Curcumin	Self-assembling	Hypoxia-responsive drug release with improved cellular uptake and the inhibition of the proliferation of HepG2 cells.	[209]
Hypoxia-responsive polymeric micelles	DOX	Self-assembly using PEG and poly-l-lysine copolymer with an azobenzene linker	High affinity to metastatic bones and response to hypoxia bone metastasis for rapid drug release with prolonged survival time.	[210]
Dual pH- and hypoxia-responsive copolymer prodrug micelles	TPZ	Self-assembling and conjugation	Higher cytotoxicity to hypoxic cancer cells.	[211]
Hypoxia-responsive nanocomplex	Double-stranded RNA	Hypoxia-cleavable polymer PEG-azo-PLL was synthesized and self-assembled into a nanocomplex	Significant in vivo antitumor effect with prolonged survival time.	[212]
Enzyme-responsive biodegradable targeted polymeric micelle	Cabazitaxel	Self-assembling	Enzyme-responsive peptides are cleavable with MMP-2. Higher cellular uptake and excellent in vivo antitumor efficacy.	[213]
Enzyme-responsive PEG peptides and star-shaped polyester NPs	Curcumin	Static electricity	MMP-responsive NPs showed higher drug loading, good biocompatibility, enhanced cellular uptake, and antitumor efficacy.	[214]
Enzyme-responsive NIR-triggered lipid polymer hybrid NPs	ICG and dichloroacetate	Chemical conjugation with self-assembling	Higher drug loading and prolonged blood circulation with synergistic photothermal/chemotherapy effects.	[215]
Hyaluronidase (HAase)- and GSH-responsive responsive nanogel	Cisplatin	Self-assembling	Stimuli-responsive nanogel possessed excellent drug- and protein-loading and intracellular delivery capabilities.	[216]

**Table 12:** Recent applications of targeted polymer-based DDSs in cancer therapy.

Delivery System	Drug	Methods	Inference	Ref.
HA-coated perfluoroalkyl polyamine NPs	siRNA	Bioactive polycationic prodrug (F-PaP) based on the anticancer polyamine analogue bisethylnorspermine modified with perfluoroalkyl moieties, following the encapsulation of siRNA, and then coated with HA	The HA-coated NPs facilitated tumor accumulation and contributed to strong tumor inhibition and the favorable modulation of the tumor immune microenvironment in an orthotopic pancreatic cancer model.	[229]
Tf-coated PLGA NPs	siRNA and DTX	DTX-conjugated PLGA copolymer and further modified with Tf peptides on the surface	Excellent antitumor effect.	[230]
Nano-PROTAC pH/GSH-responsive polymer	BRD4-targeted PROTAC (dBET6)	Self-assembly	Improved dBET6 release with remodeling of the tumor microenvironment.	[231]
RGD-decorated PLGA NPs	Cisplatin	Double emulsification method	Low systemic toxicity, high biocompatiblity, and safety, with promising anticancer effect.	[232]
GLUT1-targeted micelle	PTX and IR808	Self-assembly	Higher cytotoxicity, apoptosis rate, and metastasis inhibition both in vitro and in vivo.	[223]
LAT1-targeted PEI NPs	PTX and anti P-gp shRNA	Self-assembly and electrostatic interaction	Targeted delivery into the cells overexpressing the LAT-1 transporter.	[233]
Mitochondria-targeting polymer micelle (OPDEA-PDCA)	Dichloroacetate (DCA)	RAFT polymerization and self-assembly	Induced the secretion of PD-L1 and enhanced antitumor efficacy in combination with immunotherapy.	[234]
Antibody polymer conjugates (ADCs)	Antibody	Linker conjugation and drug conjugation	Exhibited cell targetability and selective cell killing in multiple cell lines expressing disease-relevant antigens, viz, HER2 and EGFR.	[235]

**Table 13:** Recent applications of fluorinated polymers in cancer therapy.

Delivery System	Drug	Methods	Inference	Ref.
ROS-responsive F-PEI and coated with HA	Plasmids	Thioketal-cross-linked F-PEI was further coated with HA for the delivery of plasmids	Improved the release of plasmids, reduced toxicity, and enhances the antitumor efficacy.	[241]
Fluoropolymer	Ovalbumin (OVA)	A novel fluoropolymer was developed via ring-opening polymerization and construct a fluoropolymer-based nanovaccine	Better efficacy in both pre-cancer prevention and tumor treatment and increased the proportion of CD4^+^ T and CD8^+^ T cells.	[246]
Fluorocarbon-modified chitosan (FCS)	Anti-programed cell death protein-1 (aPD-1)	Therapeutic proteins were mixed with FCS to form NPs, lyophilized with the appropriate excipients, and then filled into enteric capsules for oral administration	Promoted the transmucosal delivery and 5-fold dose oral delivery of a-PD1.	[247]
Fluorinated zwitterionic polymer-coated DNA nanoclews (FNCs)	DNA	Sequence-specific binding and coated with fluorinated zwitterionic polymer	Loaded by FNCs, an oligonucleotide can effectively silence the target miRNA when activated by NIR light and inhibit angiogenesis inside the tumor, leading to the complete ablation of the cancer.	[248]
Fluorinated PEG-PEI-coated magnetic NPs	siRNA	Heptafluorobutyryl-PEG-PEI (FPP) was first prepared and then used to coat magnetic nanoparticles (MNPs) to obtain the magnetic nanocarriers FPP@MNPs	Cellular uptake was significantly increased, and the transfection ability was increased to reach more than 90%.	[249,250]
Fluorinated gemini amphiphilic polymer (G-Fn)	CPT	Self-assembly	Excellent physical and chemical properties as well as good therapeutics.	[242]
Fluorinated covalent conjugate polymers	Sonosensitizer	Synthesized	Multifunctional nano-sonosensitizers with suppressing tumor growth ability and tumor recurrence by priming the host’s antitumor immunity.	[245]

## Abbreviations

mAbs: monoclonal antibodies; DDSs: drug delivery systems; APIs: active pharmaceutical ingredients; PDCs: polymer–drug conjugates; NPs: nanoparticles; LMW: low molecular weight; HMW: high molecular weight; HPMA: N-(2-hydroxypropyl)metharylamide; DOX: doxorubicin; PTX: paclitaxel; CPT: camptothecin; PLGA: poly (lactic glycolic acid); HA: hyaluronic acid; PEG: poly (ethylene glycol); PGA: poly (glycolic acid); PLA: poly (lactic acid); G-CSF: granulocyte-colony-stimulating factor; MPS: mononuclear phagocyte system; ECM: extracellular matrix; BBB: blood–brain barrier; TME: tumor microenvironment; CAFs: cancer-associated fibroblasts; VEGF: vascular endothelial growth factor; TNF: tumor necrosis factor; IL-6: interleukin-6; TGF-β: transforming growth factor beta; HGF: hepatocyte growth factor; FSP-1: fibroblast-secreted protein-1; SDF-1: stromal-derived factor-1; MMPs: matrix metalloproteases; IFP: interstitial fluid pressure; PSA: prostate-specific antigen; ECs: endothelial cells; GI: gastrointestinal; BMECs: brain microvascular endothelial cells; DD: degree of deacetylation; DTX: docetaxel; ROS: reactive oxygen species; ICG: indocyanine green; EPR effect: enhanced permeability and retention effect; FA: folic acid; 5-FU: 5-Fluorouracil; GSH: glutathione; PCL: polycaprolactone; PPO: polypropylene oxide; PEI: poly ethyleneimine; LAR: long-acting release; PA: palmitic acid; AS: artesunate; CQ: chloroquine; LAM: lamivudine; IRT: Irinotecan; RAL: Raloxifene; HBPs: hyperbranched polymers; 3D: three-dimensional; G0: generation 0; PPI: Polypropylene imine; PAMAM: polyamidoamine; PG: polyglycerol; CDDP: Cis-diamminodichloride Platinum; CAA: cis-Aconitic anhydride; ATRP: atom transfer radical polymerization; PDMAEMA: Poly (2-(dimethylamino) ethyl methacrylate); PNAM: Poly(N-acryloylmorpholine); PMAA: Poly(methacrylic acid); PVPA: Poly(vinyl-phosphonic acid); PVSA: Poly(vinyl-sulfonic acid); PASA: Poly(aspartic acid); PEG/PAA: poly(ethylene glycol/acrylic acid); SF: Sorafenib; H_2_S: hydrogen sulfide; PPT: Podophyllotoxin; LCST: lower critical solution temperature; PNIPAAm: Poly (N-isopropylacrylamide); Dex: dexamethasone; OA: osteoarthritis; ER: estrogen receptor; Ce6: chlorin e6; TPZ: tirapazamine; PDT: photodynamic therapy; CB: chlorambucil; TGlu: trans-glutaminase; HPR: horseradish peroxidase; Tyr: tyrosinase; PSMA: prostate-specific membrane antigen; iRGD: arginine–glycine–aspartate peptide; FR: folate receptors; Tf: transferrin; GLUT1: glucose transporter protein type 1; Met: methionine; Cys: cysteine; DCA: dichloroacetate; PFC: perfluorocarbon; OVA: ovalbumin; FCS: fluorocarbon-modified chitosan.
